# Parasite and host factors associated with malaria parasite liver stage latency

**DOI:** 10.21203/rs.3.rs-10657810/v1

**Published:** 2026-09-02

**Authors:** Gigliola Zanghi, Kim Chi Vo, Lindsay V. Clark, Sumana Chakravarty, Erika L. Flannery, Vorada Chuenchob, Yu Cao, Hardik Patel, Nastaran Rezakhani, Lucia Pazzagli, Matthew E. Fishbaugher, Sebastian A. Mikolajczak, Wanlapa Roobsoong, Jetsumon Sattabongkot, B. Kim Lee Sim, Stephen L. Hoffman, Ashley M. Vaughan, Stefan H.I. Kappe

**Affiliations:** 1 Center for Global Infectious Disease Research, Seattle Children’s Research Institute, 1916 Boren Ave, Seattle, WA 98101, USA.; 2 Center for Vaccine Development and Global Health, University of Maryland School of Medicine, Baltimore MD, USA.; 3 Research Scientific Computing, Seattle Children’s Research Institute, Seattle, Washington, United States of America; 4 Sanaria Inc., Rockville, MD 20850, USA.; 5 Novartis, Biomedical Research, Global Health, Emeryville, CA, United States of America; 6 Mahidol Vivax Research Unit, Faculty of Tropical Medicine, Mahidol University, Bangkok 10400, Thailand.; 7 Department of Pediatrics, University of Washington, Seattle, WA 98105, USA.; 8 Department of Global Health, University of Washington, Seattle, WA 98195, USA.

**Keywords:** *Plasmodium vivax*, Chesson, Liver-humanized mice, hypnozoite, relapse, primary human hepatocyte, PUF1, CCR4-NOT complex, CAF1, Spatial transcriptomics

## Abstract

*Plasmodium vivax* (*Pv*) forms dormant liver stage hypnozoites that cause relapsing malaria, a major barrier to *Pv* eradication. However, parasite-intrinsic mechanisms and host factors governing hypnozoite persistence are mostly unknown. Using purified, cryopreserved *Pv* Chesson strain sporozoites and Thai field isolates, we established reproducible liver stage infections in human hepatocyte-chimeric mice and primary human hepatocytes, generating persistent and activating hypnozoites. Transcriptomic profiling and RNA-FISH identified the RNA-binding protein *Pv*PUF1 as a conserved hypnozoite-associated marker downregulated upon activation. Liver stage expression of *Pv*PUF1 in *P. yoelii* (*Py*) induced quasi-persistent, growth-restricted liver stages *in vivo*. Comparative *in silico* analyses identified a PUF1-associated 9-mer RNA motif across *Pv* and *Py* liver stage expressed genes, linking PUF1-associated transcripts to the CCR4–NOT deadenylation complex. In addition, spatial transcriptomics revealed that hypnozoites occupy a transcriptionally quiescent host microenvironment distinct from replicating schizonts. Together, these findings identify a molecular regulator underlying hypnozoite persistence, providing a framework for therapeutic strategies targeting hypnozoites.

## INTRODUCTION

*Plasmodium vivax* (*Pv*) remains the most geographically widespread cause of human malaria outside sub-Saharan Africa, responsible for over nine million cases in 2024 and a substantial burden of recurrent clinical disease^1,2^. Infection initiates when *Plasmodium* sporozoites (SPZ) are inoculated into the host dermis by a blood-feeding female *Anopheles* mosquito^3^. SPZ enter the vasculature and migrate to the liver to invade hepatocytes^4^. The default developmental pathway of liver stage parasite is to undergo schizogony, a complex growth and replication process, which culminates in the formation and release of tens of thousands of exo-erythrocytic merozoites into the bloodstream^5^. Subsequent erythrocyte invasion enables the cyclic asexual replication responsible for all clinical morbidity and mortality, while a subset of blood stage parasites differentiates into sexual gametocytes for transmission back to the mosquito vector. *Pv*, together with *Plasmodium ovale* (*Po)* and the simian malaria parasite *Plasmodium cynomolgi* (*Pc*), belongs to a group of relapsing malaria parasites capable of forming dormant liver stages known as hypnozoites^6^. The quiescent hypnozoites exhibit reversible cellular arrest; unlike replicating schizonts, hypnozoites maintain a non-replicating but developmentally competent state that can persist within hepatocytes for weeks, months or years before activating to initiate secondary blood stage infections^6^. Thus, hypnozoites constitute a tissue reservoir of parasites that seed recurring clinical malaria infection in endemic human populations and are not directly affected by mosquito vector control and standard antimalarial drugs^7,8^.

Achieving hypnozoite elimination from the liver, termed radical cure, remains a major global health challenge. Currently, the 8-aminoquinolines primaquine and tafenoquine are the only approved drugs for hypnozoite clearance^9^. However, their clinical use is constrained by the risk of acute hemolytic anemia in individuals with glucose-6-phosphate dehydrogenase (G6PD) deficiency and pregnancy, alongside evidence of reduced tafenoquine efficacy when co-administered with antimalarial partner drugs such as dihydroartemisinin-piperaquine^10^. Developing safer, next-generation radical cure therapies is therefore critical, yet rational drug design remains fundamentally hindered by the lack of knowledge of the molecular mechanisms governing hypnozoite formation, maintenance, and activation.

Functional interrogation of *Pv* dormancy is constrained by experimental bottlenecks. In contrast to *P. falciparum* (*Pf*), the sexual stages of *Pv* lack a continuous *in vitro* culture system, precluding the routine, scalable generation of infectious SPZ. Consequently, laboratory investigations have relied predominantly on patient field isolates^11^. However, field-derived parasites are limited by logistical and biological confounding variables, including the seasonality of patient recruitment, the prevalence of polyclonal infections, and extensive inter-isolate genetic heterogeneity^11^. Furthermore, while recently developed *in vitro* liver stage platforms have allowed for preliminary transcriptomic profiling and moderate-throughput chemical screening^12–15^, long-term maintenance and the kinetic evaluation of hypnozoite activation remain technically challenging. To overcome these limitations, human hepatocyte-chimeric FRG NOD (FRGN) huHep mice have emerged as a powerful experimental platform for *Pv* liver stage development and recapitulation of relapsing blood stage infections *in vivo*^16–18^, enabling investigation of both parasite developmental programs and host parasite interactions during liver stage infection as well as interventions that target hypnozoites.

Evidence across diverse eukaryotic systems indicates that cellular quiescence is an actively maintained state governed by coordinated post-transcriptional regulatory networks rather than passive metabolic arrest^19^. In *Plasmodium* parasites, translational repression and RNA-binding proteins (RBPs) have emerged as pivotal regulators of developmental parasite stage transitions, yet the pathways associated with hypnozoite persistence remain largely undefined^20^. Recent transcriptomic studies in both *Pv* and *Pc* have identified overlapping hypnozoite-associated signatures, supporting the existence of conserved molecular programs linked to hypnozoite persistence^21,22^. Among the pathways repeatedly associated with hypnozoites are regulators of RNA stability, translational repression, and stress adaptation, suggesting that post-transcriptional control represents a central feature of hypnozoite biology^13,14,20–22^. Nevertheless, definitive mechanistic validation of these candidate pathways in *Pv* has been precluded by the lack of robust functional genetic tools and experimentally accessible model systems.

Here we used *Pv* Thai field isolates and the *Pv* Chesson strain, to investigate hypnozoite persistence. *Pv* Chesson, is phenotypically characterized by highly frequent relapse^23,24^. We generated purified, cryopreserved *Pv*Chesson SPZ, PvSPZ (Chesson) and showed that they lead to reproducible liver stage infections in both FRGN huHep mice and primary human hepatocytes, supporting hypnozoite formation, persistence, and activation *in vivo* and *in vitro*. By performing comparative transcriptomic analysis between *in vivo Pv* schizont and hypnozoite populations, we identified highly conserved hypnozoite-associated expression signatures, including upregulation of the pumilio-family RNA-binding protein (RBP) PUF1. Functional interrogation using transgenic expression of *Pv*PUF1 in *Plasmodium yoelii* (*Py*) induced persistent, growth impaired liver stages, supporting a role for *Pv*PUF1-associated regulation in hypnozoite persistence. Further, *in silico* analysis identified a *Pv*PUF1 conserved 9-mer binding motif that was found in mRNA transcripts, including mRNAs encoding components of the CCR4-NOT deadenylation complex, suggesting that RNA-binding protein-associated poly(A) deadenylation contributes to hypnozoite persistence. Finally, spatial transcriptomic profiling revealed distinct host transcriptional responses associated with schizont and hypnozoite-infected hepatocytes. Collectively, these findings provide new mechanistic insight into *Pv* hypnozoite persistence and highlight new targets for their elimination.

## RESULTS

### Purified, cryopreserved PvSPZ (Chesson) infections in FRGN huHep mice

To evaluate the infectivity of purified, cryopreserved PvSPZ (Chesson) (Extended data Fig. 1a), we used FRGN huHep mice, previously shown to support *Pv* liver stage infection and development, including schizont maturation, hypnozoite formation and hypnozoite activation^16,18^. Mice were injected intravenously with one million purified, cryopreserved PvSPZ (Chesson), a dose selected to ensure sufficient infection for quantitative analysis. Livers were collected at days 8 and 9 post-infection (p.i.), corresponding to the peak of schizont growth before exo-erythrocytic merozoite egress (Extended Data Fig. 1b). Immunofluorescence assays on liver sections confirmed the presence of both schizonts and hypnozoites in all mice, using antibodies to parasite HSP70 and the parasitophorous vacuole membrane (PVM)-expressed PvUIS4 (Figure 1a)^25^. This allowed visualization of the PVM and the characteristic PVM prominence in hypnozoites, allowing their identification^16,25^. Parasite liver stage burden following infection with cryopreserved PvSPZ (Chesson) was quantified by measuring *Pv* 18S rRNA levels in FRGN huHep livers (Figure 1b). This showed a significant liver stage infection, comparable to that obtained at the same PvSPZ inoculum using fresh *Pv* Thai SPZ obtained from mosquitoes that were infected by feeding on infected human blood^11^. Hypnozoite and liver stage schizont numbers were quantified across infected liver lobes by immunofluorescence microscopy (Figure 1c). Across biological replicates, the hypnozoite:schizont ratio was highly reproducible, averaging 40%:60% (Figure 1d). To assess egress of merozoites from hepatocytes, blood from infected mice was tested at day 9 p.i. by *Pv* 18S qRT-PCR^18^. All samples were qPCR positive and showed comparable levels to infections initiated with fresh *Pv* Thai SPZ (Figure 1e). To evaluate relapse dynamics and confirm that *Pv* Chesson hypnozoites formed by cryopreserved PvSPZ (Chesson) were capable of activation, FRGN huHep mice were infected with doses of 1 million, 100,000 and 15,000 PvSPZ (Chesson) and monitored daily between days 9–24 p.i. (Figure 1f). Since the *Pv* Chesson strain is characterized by short latency^23,26^, this three-week window was chosen to capture the first relapse events. *Pv* 18S RNA was detected after merozoite egress from primary liver stage schizonts at day 9 p.i. in the blood of all groups. A qPCR signal was next seen between days 17–20 p.i., regardless of inoculum size (Figures 1f). Since the infected FRGN huHep mice were not repopulated with human reticulocytes and *Pv* cannot invade murine erythrocytes, the detected 18S signal at day 17–20 p.i. likely originated from merozoites released from activated hypnozoites that formed secondary liver stage schizonts. The *Pv* Chesson relapse pattern observed in the FRGN huHep mouse model is similar to observations we have made using *Pv* Thai field isolates and aligns with predictions indicating that *Pv* Chesson and *Pv* Thai field isolates share similar relapse phenotypes^7,18^. Together, these findings demonstrate that purified, cryopreserved PvSPZ (Chesson) establish robust liver stage infections with schizonts and hypnozoites, the latter capable of activation and relapse.

### Infection and liver stage maturation of *Pv* Chesson across primary human hepatocyte donors.

We next evaluated the *in vitro* infection of purified, cryopreserved PvSPZ (Chesson) using primary human hepatocytes from four independent donors (BGW, ESV, HTL and ONR, Extended data Figure 2a). Hepatocytes were seeded in 384-well plates at 18 × 10^3^ cells per well and infected at a multiplicity of infection (MOI) of 1:1. Cultures were fixed at days 3, 5, and 7 days p.i. and analyzed by immunofluorescence assay to quantify total liver stage parasites (Figure 2a) and distinguish schizonts from hypnozoites. All hepatocyte donors supported PvSPZ (Chesson) infection of hepatocytes, although infection frequency (Figure 2a) and parasite development varied across donors (Figure 2b and Extended data Fig. 2b), consistent with previous observations^27^. Across all donors and time points, liver-stage populations consisted of approximately 70% schizonts and 30% hypnozoites (Figure 2b–c and Extended Data Fig. 2b-c). Despite a similar hypnozoite ratio across donors, the magnitude and quality of infection differed markedly. Donors BGW and ESV supported the highest and most reproducible liver-stage infections, with over 100 parasites per well (0.56% infection rate) (Figure 2a and 2d). Donor ONR yielded the highest overall liver-stage numbers (Figure 2a), but schizonts were small at day 7 p.i. (Figure 2d,e). Hepatocytes from donor HTL were the least supportive of infection, showing a decline in liver stage numbers over time (Figure 2a) with small schizonts at day 7 p.i. (Figure 2d and 2e). Liver stage size distributions across donors displayed the expected bimodal profile, reflecting small hypnozoites and large schizonts (Figure 2d and 2e). This was validated by detailed immunofluorescence inspection of the anti-PvUIS4 antibody, which clearly distinguished hypnozoites with the characteristic PvUIS4 prominence from schizonts in all donors (Figure 2d). Together, these results show that purified, crypreserved PvSPZ (Chesson) are infectious *in vitro*, generating liver stage schizonts and hypnozoites. However, *Pv* liver stage development was strongly influenced by donor-specific hepatocyte factors, resulting in substantial variation in infection efficiency and schizont maturation (Figure 2d and 2e). Based on these data, the ESV hepatocyte donor was selected for further experiments since liver stage numbers, and schizont maturation were optimal.

### PUF1 expression is associated with hypnozoites by RNA-FISH

To characterize hypnozoites at the molecular level, bulk RNA sequencing (RNA seq) was performed on FRGN huHep mice infected with PvSPZ (Thai). This analysis relied on Thai SPZ since it was carried out prior to PvSPZ (Chesson) becoming available. Hypnozoite enrichment was achieved via administration of a phosphatidylinositol 4 kinase [PI(4)K] inhibitor from days 4 to 7 p.i. to selectively clear replicating schizonts while preserving hypnozoites^28^ and untreated animals served as mixed liver stage population (schizont and hypnozoite) controls. At day 8 p.i., treatment efficacy was evaluated by immunofluorescence microscopy of a single liver lobe from each mouse to quantify parasite burden and verify schizont clearance and retention of the hypnozoite population (Figure 3a). Total RNA was extracted from infected livers and enriched for *Pv* transcripts using custom tiling baits spanning the *Pv* genome^12,13,29^. Bulk RNAseq analysis of probeenriched samples from PI(4)K treated and untreated FRGN huHep mice revealed a transcriptional signature that distinguished hypnozoites from schizonts (Figure 3b and Table 1). Initial assessment of the mixed liver stage population control samples confirmed a predominance of transcripts associated with active replication, verified by the expression of merozoite surface protein 1 (*PvMSP1*) consistent with mature schizont development at day 8 p.i. (Figure 3b and Table 1). In contrast, comparative transcriptomic profiling of the PI(4)K-treated mice revealed a restricted subset of differentially expressed genes. (Extended Data Fig. 3a). These hypnozoite-enriched transcripts exhibited significant gene ontology (GO) enrichment for pathways involved in post-transcriptional regulation, including cytoplasmic stress granules, supramolecular complexes, and ribonucleoprotein granules, which mediate mRNA storage, stability, and translational repression (Extended Data Fig. 3b). Notably, the most highly upregulated transcript within the hypnozoite population was the pumilio family RNA-binding protein 1 (*Pv*PUF1) (Figure 3b, Extended data Figure 3a and Table 1). Elevated *Pv*PUF1 expression in hypnozoites has been independently observed in previous bulk and single-cell transcriptional studies profiling early liver stages of relapsing malaria parasites isolated from *in vitro* cultures^12–14^. To determine whether *Pv*PUF1 expression could serve as a molecular marker distinguishing hypnozoites from schizonts, we used RNA fluorescence in situ hybridization (RNA-FISH) in primary human hepatocytes infected with PvSPZ (Chesson) and PvSPZ (Thai field) (Figure 3c and Extended data Figure 3c). We also used RNA-FISH to detect the *PvLISP2* transcript, which is expressed during liver-stage schizogony in *Pc* and *Pv*^30,31^, together with PvUIS4 immunostaining to delineate the parasite PVM and DAPI for nuclear visualization (Figure 3c and Extended Data Fig. 3c). As expected, schizonts consistently expressed both *PvLISP2* transcript and protein (Figure 3c and Extended Data Fig 3c and 3d). In contrast, distinct *Pv*PUF1 RNA foci were observed exclusively in hypnozoites and remained undetectable in schizonts across primary human hepatocytes infected with both PvSPZ (Chesson) and PvSPZ (Thai field) (Figure 3c and Extended Data Fig. 3c). Quantitative analysis across independent experimental replicates confirmed the selective enrichment of *PvPUF1* RNA foci in all morphologically defined hypnozoites from both parasite lines (Extended Data Fig. 3e). Interestingly, hypnozoites from Thai field isolates exhibited less detectable *PvLISP2* transcript despite retaining detectable LISP2 protein, whereas *Pv* Chesson hypnozoites displayed robust LISP2 detection at both the transcript and protein levels (Extended Data Fig. 3d,e).

To assess whether *Pv*PUF1 signal persisted upon hypnozoite activation, infected cultures were maintained to day 17 p.i., a time point characterized by the presence of hypnozoites and newly formed secondary schizonts (Figure 3d and 3e). Activated liver stages displayed intense *LISP2* signal and undetectable *Pv*PUF1 expression, a phenotype equivalent to primary schizonts (Figure 3f). Conversely, quiescent hypnozoites selectively retained *Pv*PUF1 expression (Figure 3f). Collectively, these data demonstrate that *Pv*PUF1 serves as a reliable marker of the hypnozoite state and is downregulated upon activation and the subsequent transition to schizogony.

### Expression of *PvPUF1* in *P. yoelii* induces growth-arrested, quasi-persistent liver stages.

Direct interrogation of *PvPUF1* function is limited in the absence of *Pv* transgenesis. Consequently, to model *PvPUF1* function during liver stage development, a transgenic *Py* line was generated to ectopically express *Pv*PUF1 under the control of the liver stage specific *UIS4* promoter and terminator regulatory elements, as previously described^22^. The *PvPUF1* coding sequence was integrated into the dispensable *PyP230p* locus by CRISPR/Cas9 homologous recombination and fused at the C-terminus to mCherry to enable visualization of parasite development to create *Py*^*UIS4*^*Pv*PUF1^*mCherry*^ (*Py*^*Pv*PUF1^) (Extended Data Fig. 4a). Integration of the expression cassette was confirmed by PCR and sequencing (Extended Data Fig. 4a).

The *Py*^*Pv*PUF1^ line was evaluated across the parasite life cycle to determine whether ectopic *PvPUF1* expression affected parasite fitness. Asexual parasitemia, exflagellation rates, mosquito oocyst burdens, and salivary gland sporozoite numbers were comparable between *Py*^*Pv*PUF1^ and wild type (*Py*WT) parasites (Extended Data Fig. 4b).

The impact of *Pv*PUF1 expression on liver stage development was subsequently evaluated in HepG2-CD81 cells infected with SPZ. At 44 hours p.i., *Py*^*Pv*PUF1^ parasites displayed significantly impaired intrahepatic growth relative to the *Py*WT control, characterized by a marked reduction in overall parasite size (Figures 4a and b). Immunofluorescence microscopy revealed extensive retardation in organellar development, including stunted development of the apicoplast, endoplasmic reticulum, and mitochondrion, alongside an absence of the mature schizont marker MSP1 (Extended Data Fig. 4c and 4d). Despite the reduction in parasite size, the number of infected hepatocytes remained comparable between *Py*^*Pv*PUF1^ and the *Py*WT parasites, indicating that the *Py*^*Pv*PUF1^ liver stages were viable and not cleared.

To assess liver stage development *in vivo*, BALB/cByJ mice and Swiss Webster mice (data not shown), were intravenously co-infected with a total of one million SPZ consisting of 500,000 *Py*^*Pv*PUF1^ and 500,00 *Py*WT parasites, allowing for a direct comparison of parasite development within the same host environment (Figure 4c). Transgenic parasites were differentiated from *Py*WT controls by mCherry expression. Quantitative imaging analysis performed at day 2 p.i. identified two parasite populations within the *Py*^*Pv*PUF1^ line: 70% of parasites consisted of markedly small liver stages, whereas the remaining 30% were larger schizont-like parasites that nevertheless remained smaller than *Py*WT liver stages (Figure 4d and 4e).

Temporal progression of these populations was monitored via immunofluorescence microscopy of infected liver tissues collected at days 3, 4, 5, and 6 days p.i. (Figure 4f). Size analysis across the time course showed that the larger *Py*^*Pv*PUF1^ parasites were no longer detectable after day 3 p.i., whereas the smaller liver stages persisted through day 6 p.i. (Figure 4f–g). Quantification of the persistent small liver stages demonstrated that the number of infected hepatocytes containing *Py*^*Pv*PUF1^ parasites remained stable across time points (Figure 4h). Analysis of blood stage patency showed that *Py*WT parasites became patent by day 3 p.i. following either intravenous *Py*SPZ inoculation or by mosquito bite transmission, whereas mice infected with *Py*^*Pv*PUF1^ SPZ exhibited a consistent 24-hour delay in patency regardless of the inoculation route (Figure 4i). Collectively, these findings indicate that *Pv*PUF1 expression induces a bimodal liver stage phenotype characterized by a subset of a developmentally competent population alongside a persistent population of smaller liver stages that remain growth-arrested within hepatocytes beyond the normal two day duration of *Py* liver stage development.

### Comparative motif analysis identifies conserved PUF1-associated transcripts across *P. vivax* and *P. yoelii*.

To investigate the molecular mechanisms underlying the persistent liver stage phenotype induced by *Pv*PUF1 expression in *Py*, we first characterized the RNA-binding specificity of *Pv* and *Py* PUF1 and then identified putative PUF1-binding motifs in the liver-stage transcriptomes of both *Pv* and *Py*. PUF1 belongs to the conserved Pumilio family of RBP, which regulate mRNA stability and translation through sequence-specific interactions with target transcripts. Pumilio proteins typically recognize short sequence motifs through a conserved RNA-binding domain composed of eight tandem Pumilio repeats^32^.

To define the RNA-binding specificity of *Pv*PUF1, AlphaFold structural predictions (extended data Figure 5a and 5b) of *Pv*PUF1 and *Py*PUF1 were combined with multiple sequence alignments of PUF1 orthologues from *Plasmodium* species, *Toxoplasma gondii*, *Drosophila melanogaster*, *Saccharomyces cerevisiae*, and humans (Extended Data 1). Analysis of the 24 amino acid residues predicted to interact with the RNA and influence binding specificity revealed complete conservation between *Pv*PUF1, *Py*PUF1, and the well-characterized *S. cerevisiae* PUF4 protein^33^ (Extended Data 1). These findings predict that both *Pv*PUF1 and *Py*PUF1 recognize the canonical 9-mer Pumilio-binding motif UGUANNNUA (Figure 5a). Although some of the conserved residues are not represented in the RNA-recognition code derived from mutagenesis studies of human PUF proteins^34,35^, the RNA-binding specificity of *S. cerevisiae* PUF4 has been experimentally established, supporting this prediction^33^.

We next examined the distribution of sequence variants matching the predicted PUF1-binding consensus across annotated 5′UTRs, 3′UTRs, and coding sequences within the *Pv* liver stage transcriptome. Multiple motifs were significantly enriched relative to random expectation (FDR < 0.05), including ten motifs in *Pv* 3′UTRs, ten in 5′UTRs, and five in coding regions (extended Data Figure 5c). Among the significantly enriched motifs, members of the canonical Pumilio-like UGUAWWWUA family and UGUACAUUA were prominently represented (Table 2).

To determine whether any of the enriched motifs were associated with hypnozoites, we identified transcripts whose abundance was upregulated in *Pv* hypnozoites vs. schizonts (Figure 5b). Among all significantly enriched motifs, only the 3′UTR motif UGUACAUUA was represented in hypnozoite upregulated transcripts (Figure 5b). Genes containing this motif exhibited significantly higher log_2_ fold-change values than genes lacking the motif (Figure 5b and Extended Data Figure 5d). In contrast, neither UGUAWWWUA-family motifs nor UGUACAUUA motifs within 5′UTRs were associated with hypnozoite-enriched expression (Table 2).

To determine whether transcripts containing similar motifs were associated with the growth-restricted liver-stage phenotype induced by *Pv*PUF1 expression in *Py*, we performed an equivalent analysis using the bulk RNA-seq transcriptome generated for this study from fluorescence-activated cell-sorted GFP-positive *Py*-infected hepatocytes at 36 h p.i. from both *in vitro* and *in vivo* infections (Table 1). Consistent with the *Pv* analysis, multiple motifs were significantly enriched relative to random expectation, including eight motifs in 3′UTRs, seven motifs in 5′UTRs, and one motif in coding regions (Extended Data Fig. 5c). Orthology analysis identified 3,511 genes expressed in the liver stage transcriptomes of both species (Extended Data Fig. 5e). Among these, 2,590 orthologous gene pairs contained UGUAWWWUA-family motifs and/or the UGUACAUUA motif in either *Pv*, *Py*, or both species (Table 2). This included 496 orthologous gene pairs containing motifs in both species (1.5-fold more than expected by chance), 1,941 genes containing motifs exclusively in *Py*, and 153 genes containing motifs exclusively in *Pv* (Figure 5c). The remaining 921 orthologs lacked either motif in both species. Mapping of motif-containing orthologs revealed that conserved PUF1-associated motifs were distributed across coding sequences, 5′UTRs, and 3′UTRs in both *Pv* and *Py* (Figure 5d).

GO enrichment analysis revealed distinct functional signatures among motif-containing transcripts unique to each species and those conserved between them (Figure 5e). Motif-containing transcripts exclusive to *Pv* were primarily enriched for pathways associated with transcriptional and post-transcriptional regulation, including RNA binding, regulation of transcription by RNA polymerase II, and U2 snRNP-associated functions, which could be linked to hypnozoite persistence. In contrast, *Py* exclusive transcripts were enriched for pathways linked to protein homeostasis and translation, including ribosome binding and the eukaryotic translation initiation factor 2B complex. Notably, the conserved set of motif-containing orthologs shared between *Pv* and *Py* was enriched for DNA replication, DNA-directed DNA polymerase activity, DNA replication factor C complex, and mRNA binding, suggesting that PUF1-associated transcripts participate in DNA replication and developmental progression during liver-stage development. Notably, transcripts encoding components of the CCR4–NOT complex were also enriched within the conserved motif-containing transcript set (Table 2). Given the established role of CCR4–NOT-mediated deadenylation in post-transcriptional regulation, we next examined expression of the complex components across *Pv* and *Py* liver stage transcriptomes (Figure 5f). Multiple members of the CCR4–NOT complex were expressed at low levels in replicating *Py* and *Pv* schizonts, whereas several members showed increased transcript abundance in hypnozoites (Figure 5f). The catalytic deadenylase subunit of the CCR4–NOT complex, CAF1, which mediates poly(A)-tail degradation and contains a predicted UGUACAUUA *Pv*PUF1-binding motif, was upregulated in *Pv* hypnozoites relative to schizonts (Figure 5f). Together, these results support an association between *Pv*PUF1 expression, RNA regulatory pathways, and hypnozoite-associated transcriptional programs.

### Spatial transcriptomics reveals distinct host liver microenvironments in schizont and hypnozoite infected liver tissue

Having identified parasite-intrinsic regulatory mechanisms associated with hypnozoite persistence, we next investigated whether parasite developmental state is associated with distinct local host transcriptional programs. To address this, we performed GeoMx digital spatial transcriptomics on regions containing *Pv* liver stage schizonts or hypnozoites. Immunofluorescence assays were carried out on livers from FRG NOD huHep mice infected with PvSPZ (Chesson) and assessed at day 8 p.i. Sections were probed with antibodies against cytokeratin (CK18/8) to visualize hepatocyte membranes, fumarylacetoacetate hydrolase (FAH), to identify human hepatocytes and to verify intracellular parasite localization within human cells^36^, and HSP70 to visualize liver stage parasites. Regions of interest (ROIs) were systematically selected based on parasite morphology to capture developing schizonts (three ROIs), hypnozoites (six ROIs), and uninfected hepatocytes (three ROIs) (Figure 6a). These ROIs were then profiled using the GeoMx Whole Transcriptome Atlas (WTA), to capture and sequence ~18,000 human transcripts within the ROIs (Extended Data Figure 6a and Table 3). Because each designated ROI encompasses the infected hepatocyte along with its immediate neighboring parenchymal cells, these data reflect the collective transcriptomic state of the local cellular microenvironment rather than the isolated infected cell. Unsupervised clustering and dimensional reduction analyses demonstrated that schizont containing microenvironments segregated into a distinct and cohesive transcriptional cluster, clearly separated from both hypnozoite-infected and uninfected tissue areas (Extended Data Figure 6b). Quantification of differentially expressed genes (Figure 6b) indicated that schizont containing ROIs undergo broader transcriptional remodeling than hypnozoite containing ROIs relative to uninfected tissue, indicating that replicating parasites significantly reshape their local cellular microenvironment. Conversely, hypnozoite containing ROIs displayed fewer transcriptional changes compared to uninfected ROIs, consistent with a more inert niche. Volcano plots (Figure 6c and Table 3) further illustrate these contrasts. Consistent with previous single-cell studies of *Pv in vitro* liver stage infections^13,14^, schizont-containing regions showed broad upregulation of genes involved in metabolic activation, mitochondrial function, translation, and stress responses, including multiple ribosomal proteins, oxidative phosphorylation components, and metabolic enzymes (Figure 6c and extended data Fig 6c). This transcriptional signature reflects the high anabolic demand associated with active schizont growth, nuclear replication and merozoite formation. Only a small set of transcripts was significantly modulated in hypnozoite-containing ROIs versus uninfected ROIs, with modest fold changes, underscoring the quiescent character of hypnozoite infections and/or the dominance of uninfected hepatocytes in the sampled regions. In the hypnozoitecontaining ROIs we observed a modest modulation of innate-immune genes, metabolic remodeling, post-transcriptional/proteostasis regulators and survival-associated transcripts (Figure 6c and extended data Fig 6c). These results collectively reflect the minimally perturbed, hypnozoite-containing hepatic niche. Interestingly, direct comparison of hypnozoite-containing and schizont-containing ROIs did reveal divergent host-pathway remodeling (Figure 6d and Extended data Figure 6c). Schizont-containing regions showed pathways related to translation and ribosome biogenesis, oxidative phosphorylation, mitochondrial metabolism, RNA processing, and stress and apoptosis signaling, reflecting the high biosynthetic and energetic demands of a developing schizont. In contrast, hypnozoite-containing ROIs displayed selective enrichment of pathways associated with lipid handling, autophagy regulation, nuclear receptor signaling, and basal immune homeostasis, and reduced activation of anabolic, proliferative, and inflammatory pathways, indicating that hypnozoite-containing ROIs maintain a metabolically restrained and immunologically quiescent microenvironment. Together, these microenvironment pathway distinctions highlight that while replicating parasites extensively impact and remodel the surrounding hepatic niche to support cellular growth, hypnozoites occupy a minimally perturbed, immunologically quiescent microenvironment optimized for long term persistence.

## DISCUSSION

Despite decades of investigation, the molecular mechanisms governing hypnozoite persistence in *Pv* have remain largely unknown due to the limited availability of PvSPZ, the difficulties associated with the genetic manipulation of *Pv*, and the lack of experimental systems that faithfully recapitulate human liver stage biology. Here, we integrated complementary *Pv* infection models spanning cryopreserved PvSPZ (Chesson) and PvSPz (Thai field isolates) with FRGN huHep mice, primary human hepatocyte cultures, RNA-FISH, genetic manipulation in the rodent malaria parasite *Py*, and spatial transcriptomics. We show that purified, cryopreserved PvSPZ (Chesson) retain full developmental competence, generating both schizonts and hypnozoites at physiologically relevant ratios while preserving the capacity for activation and blood stage transition, and validate these findings using Thai field isolates. Together, these approaches overcome longstanding technical barriers to *Pv* liver-stage research and establish an integrated experimental platform for interrogating parasite-intrinsic mechanisms alongside parasite-driven remodeling of the host hepatic microenvironment during hypnozoite persistence.

Using PI(4)K inhibitor-based enrichment in FRGN huHep mice, we defined the *in vivo* transcriptional landscape of hypnozoites and identified *Pv*PUF1, a Pumilio-family RNA-binding protein, as the most upregulated transcript in hypnozoite-enriched samples. RNA-FISH further demonstrated that *Pv*PUF1 expression was restricted to hypnozoites across both Chesson and Thai field isolates and was lost upon hypnozoite activation, supporting its association with the dormant state. The enrichment of *Pv*PUF1 transcripts in hypnozoites across both laboratory and field isolates, together with its recurrent detection across independent *Pv* single-cell transcriptomic studies^12–14^, identifies *Pv*PUF1 as a conserved marker associated with hypnozoite persistence.

Functional evidence supporting a role for *Pv*PUF1 in hypnozoite persistence emerged from heterologous expression in *Py*, which generated two distinct liver-stage populations, resulting in a striking bimodal size distribution closely resembling the coexistence of hypnozoite-like and schizont-like populations observed during *Pv* liver-stage development. Importantly, the persistent phenotype observed following *Pv*PUF1 overexpression has not been reported for other liver-stage RNA-binding proteins perturbations^22^. Overexpression of *Pv*PUF2 in rodent malaria parasites produces no detectable liver stage phenotype, indicating that not all Pumilio-family proteins exert equivalent developmental control during liver stage growth^22^. By contrast, heterologous expression of the recently identified RNA binding proteins *Pv*IESI-1 and *Pv*IESI-2 arrests schizogony but ultimately results in parasite death rather than persistence^22^. Notably, *Pv*IESI-2 RNA-binding motifs are enriched within the *Pv*PUF1 transcript^22^, raising the possibility that *Pv*IESI-2 acts upstream of *Pv*PUF1. Together, these findings support a model in which *Pv*IESI-2 promotes hypnozoite formation through regulation of *Pv*PUF1, whereas *Pv*PUF1 functions in both the establishment and maintenance of the persistent state.

Pumilio-family RBP are evolutionarily conserved post-transcriptional regulators that govern developmental transitions, cellular plasticity, and stress adaptation across eukaryotes through the selective repression of target transcripts^37^. Across multiple independent systems, including stem cell maintenance, germline specification, and cellular quiescence, PUF proteins function as molecular gatekeepers that stabilize non-proliferative states while preserving developmental competence^33^. *Plasmodium* parasites encode three conserved PUF proteins whose functions have diverged across developmental stages and species^38,39^. *Pf*PUF1 is required for the maintenance of mature female gametocytes^40^, whereas *Pf*PUF2 represses transmission-stage transcripts and, in rodent malaria parasites, maintains salivary gland sporozoites in a latent state until transmission^41,42^. By contrast, PUF3 appears to fulfill an essential housekeeping role in ribosome biogenesis rather than developmental regulation^39^. Together, these studies establish PUF proteins as central regulators of developmental fate throughout the *Plasmodium* life cycle, yet no member of this family had previously been implicated in hypnozoite persistence. Our findings identify *Pv*PUF1 as a regulator associated with hypnozoite persistence, extending the role of PUF-mediated post-transcriptional regulation to the dormant liver stage.

The conservation between the predicted *Pv*PUF1 recognition sequence and the canonical binding motif of *Saccharomyces cerevisiae* PUF4 further suggests that hypnozoite persistence may exploit an evolutionarily conserved mechanism of RNA-mediated cellular quiescence^33^. Consistent with this, motif enrichment analysis identified a conserved set of orthologous transcripts containing related 9-mer motifs in both *Pv* and *Py* liver stages. Functional enrichment analysis of these motif-containing transcripts identified processes associated with RNA metabolism and DNA replication, suggesting that *Pv*PUF1 regulates a network of transcripts involved in liver-stage developmental progression.

Our in silico analyses identified *Pv*PUF1 recognition sequences within the 3′UTRs, 5′UTRs, and coding regions of transcripts expressed during the liver stage, indicating that *Pv*PUF1 may regulate target transcripts through multiple sequence contexts. Interestingly, the *Pv*PUF1 transcript itself contains a predicted *Pv*PUF1-binding motif within its 5′UTR. Although PUF proteins canonically recognize motifs within 3′UTRs, recent studies have shown that PUF proteins can also bind target sequences located within the 5′UTRs and coding regions^37,43^, raising the possibility that *Pv*PUF1 may be subject to autoregulation. The enrichment of multiple CCR4–NOT-associated transcripts within the motif-containing gene set, together with the increased abundance of the catalytic deadenylase CAF1 in hypnozoites, further implicates post-transcriptional regulation in hypnozoite persistence. Notably, CAF1 transcripts were absent from *Pf* Day 2 liver-stage trophozoites^29^, suggesting that CAF1 expression is associated with hypnozoite biology rather than early liver-stage development. In diverse eukaryotic systems, PUF-family proteins interact with the CCR4–NOT complex to regulate mRNA stability and translational control^37^. Together with previous studies implicating both PUF-family proteins and the CCR4–NOT complex in post-transcriptional regulation during *Plasmodium* gametocyte development^40,44^, our findings raise the possibility that *Pv*PUF1 functions within a conserved RNA regulatory network adapted to maintain the hypnozoite state.

Although our findings identify parasite-intrinsic RNA regulation as a key feature of hypnozoite persistence, this phenotype is ultimately established within the host hepatic microenvironment. We therefore investigated how the parasite developmental state is associated with local host transcriptional responses. Spatial transcriptomic analyses revealed distinct hepatic microenvironments associated with schizonts and hypnozoites. Regions containing schizonts exhibited strong enrichment of pathways involved in oxidative phosphorylation, ribosome biogenesis, mitochondrial metabolism, protein synthesis, and vesicular trafficking, consistent with the intense biosynthetic and energetic demands of replicating parasites. In contrast, hypnozoite-associated regions displayed a comparatively quiescent transcriptional profile characterized by reduced anabolic and stress-response signaling, altered lipid metabolism, and relative immunological quiescence. These findings suggest that hypnozoites reside within a metabolically restrained and immunologically permissive hepatic niche that may facilitate long-term persistence.

Collectively, our findings identify *Pv*PUF1 as a conserved regulator associated with hypnozoite persistence and implicate an evolutionarily conserved post-transcriptional regulatory network in liver stage dormancy. A limitation of the present study is that the molecular mechanisms underlying hypnozoite persistence were investigated in immunodeficient FRGN huHep mice and *in vitro* primary human hepatocyte cultures, precluding assessment of how host immune responses contribute to hypnozoite formation, maintenance, and activation. Nevertheless, the persistence of *Pv*PUF1-overexpressing *Py* parasites in two independent immunocompetent mouse strains suggests that *Pv*PUF1 promotes a parasite state that is not readily eliminated by the host immune system. Likewise, defining the direct RNA targets of *Pv*PUF1, establishing its bona fide RNA-binding motif(s), and elucidating its interactions with the CCR4–NOT complex will be essential to establish the molecular basis of its regulatory function. Addressing these questions will provide important mechanistic insight into the molecular pathways governing hypnozoite persistence and activation. Together, these findings provide a conceptual framework for understanding hypnozoite persistence and identify new avenues for the rational development of therapies targeting dormant liver-stage malaria parasites.

## MATERIALS AND METHODS:

### Ethical oversight

All animal studies were conducted in accordance with applicable institutional and NIH Office of Laboratory Animal Welfare (OLAW) standards. Mouse experiments were performed under OLAW Animal Welfare Assurance #D16–00119 and protocols approved by the Institutional Animal Care and Use Committee (IACUC) at the Center for Global Infectious Disease Research, Seattle Children’s Research Institute (SCRI). *P. yoelii* experiments were conducted under protocols SK00666. FRGN huHep mice used for *P. vivax* liver-stage studies were maintained and handled under the corresponding SCRI IACUC-approved protocol AV00480. Mice were housed under specific pathogen-free conditions with a 12-h light/12-h dark cycle at 72 °F and 45% relative humidity.

Studies involving *Saimiri boliviensis* were performed at BIOQUAL, Inc. Animals were obtained from the MD Anderson Cancer Center, Texas, and housed and subjected to experimental procedures at BIOQUAL. BIOQUAL maintains a centralized animal care and use program registered with the U.S. Department of Agriculture (USDA) and accredited by AAALAC International. Animals were monitored at least twice daily by trained animal care and technical staff for general health and signs of pain or distress, with veterinary evaluation and treatment provided when necessary. Telazol (4.0–6.0 mg/kg) or ketamine (5–20 mg/kg) was used for anesthesia during blood collection and dosing procedures. Blood collection volumes and schedules followed the general principles outlined in A Good Practice Guide to the Administration of Substances and Removal of Blood, Including Routes and Volumes (J. Appl. Toxicol. 21, 15–23, 2001).

### Mosquito Rearing and Plasmodium vivax sporozoite (PvSPZ) Chesson Production

*Anopheles stephensi* mosquitoes were reared and maintained at Sanaria using standard methods used for producing all of Sanaria’s *Plasmodium falciparum* (Pf) sporozoite products^45–47^. *Saimiri boliviensis* non-human primates (NHPs) were infected with asexual erythrocytic stage *Plasmodium vivax* (Pv) (Chesson) parasites. When gametocytes were present, blood was obtained and fed to mosquitoes through a membrane feeder. 14–17 days later, PvSPZ (Chesson) were extracted from the mosquitoes by dissection of salivary glands, purified, formulated and cryopreserved in liquid nitrogen vapor phase (LNVP) as described^48^.

### *P. vivax* Thailand SPZ isolation

To obtain PvSPZ (Thai), mosquitoes were fed on *P. vivax* infected blood obtained from patients seeking treatment for *P. vivax* malaria at malaria clinics along the Thai–Myanmar border. Infected mosquitoes were shipped to Seattle Children’s Research Institute where SPZ were obtained by microdissection of the mosquitoes’ salivary glands 14–16 days after the blood meal. SPZ were collected into cold Schneider’s insect media (Sigma Aldrich). Salivary glands were crushed and SPZ were enumerated using a hemocytometer.

### Mosquito Rearing and *P. yoelii* SPZ Production

*Anopheles stephensi* mosquitoes were reared and maintained according to the standard procedures described in the Methods in Anopheles Research (MR4) manual. Mosquitoes were housed in a secure ACL1 insectary at 27 °C and 75% relative humidity under a 12 h light/12 h dark photoperiod. Cotton pads soaked in 8% (w/v) dextrose supplemented with 0.05% (w/v) para-aminobenzoic acid (PABA) were provided daily.

Mosquitoes were infected by feeding on Swiss Webster (SW) mice infected with either *Py*WT or the transgenic *Py*^*Pv*PUF1^ parasite line. Midguts were dissected 7 days after the infectious blood meal to assess mosquito infection by evaluating oocyst development. Salivary gland sporozoites were harvested in Schneider’s insect medium 14–15 days after the infectious blood meal.

### Mice

Female FRGN huHep mice (>4 months of age) were purchased from Yecuris, Inc. Human hepatocyte repopulation was confirmed by measuring serum human albumin levels, and only animals with >70% human hepatocyte repopulation (>4 mg/mL human serum albumin) were included in the study, as previously described^36^. Female BALB/cByJ mice were used for *Py* liver-stage infection experiments and hepatocyte isolation. Female Swiss Webster (SW) mice were used for the recovery, propagation, and cloning of transfected *Py* parasites. All mice were maintained under specific pathogen-free conditions with a 12 h light/12 h dark cycle at 72°F and 45% relative humidity in the Center for Global Infectious Disease Research animal facility at Seattle Children’s Research Institute (SCRI). All animal procedures were performed in accordance with protocols approved by the Seattle Children’s Research Institute Institutional Animal Care and Use Committee (IACUC) under protocols AV00480 and SK00666.

### *In vivo* infections

Infection of FRGN huHep mice with PvSPZ. Immediately after isolation or thawing, one million PvSPZ were injected intravenously into FRGN huHep mice. To prevent bacterial coinfections, mice were injected intraperitoneally with 1000 Units Penicillin and 1mg Streptomycin (Sigma Aldrich) every other day after the infection. Infection of mice with *P. yoelii* sporozoites. Salivary gland sporozoites were isolated from *Anopheles stephensi* mosquitoes infected with either *Py*WT or the transgenic *Py*^*Pv*PUF1^ parasite line 14–15 days after the infectious blood meal. For liver-stage infectivity and time-course analyses, BALB/cByJ mice were co-infected by intravenous retro-orbital injection with an equal mixture of *Py*WT and *Py*^*Pv*PUF1^-mCherry sporozoites (5 × 10^5^ of each parasite line; 1 × 10^6^ total sporozoites per mouse). For patency experiments, Swiss Webster (SW) mice were infected with 10000 *Py*WT or *Py*^*Pv*PUF1^ sporozoites either by intravenous retro-orbital injection or by mosquito bite.

### 18S rRNA qRT-PCR quantification of parasite load

Per sample, 50μl blood was drawn and immediately added to 1ml NucliSENS Lysis buffer (bioMérieux, Marcy-l’Étoile, France) and frozen at −80°C. The samples were processed and analyzed for presence of Plasmodium 18S rRNA using a pan-Plasmodium probe as previously described (Seilie et al., 2019). This assay has a limit of detection of 20 parasites/ml blood or 148,000 18S RNA copies/ml blood.

### *In vitro* infection

Primary human hepatocyte (PHH) cultures were prepared from four different batches of human hepatocytes (BGW, ESV, HTL and ONR) following the manufacturer’s instructions. Briefly, cryopreserved PHH (BioIVT International) were thawed and resuspended in seeding medium according to the manufacturer’s instructions. Cells were seeded in 384-well plates previously coated with rat tail collagen I at a density of 18000 cells per well, and after cell attachment. Three days after plating cells were infected with PvSPZ (Chesson). Plates were centrifuged at 2,000 rpm for 10 min at RT to facilitate parasite settling onto the cells. Medium was changed 3 hr post-infection; medium was replaced every other day. Cultures were stopped on days 3, 5, 8, 11,13 or 17 post-infections by fixing with paraformaldehyde 4%. Parasite numbers within hepatocytes were assessed and counted by immunofluorescence microscopy using anti-PfHSP70 and anti-PvUIS4 antibodies as previously described^25^. Similarly, HepG2-CD81 cells were seeded at a density of 50,000 cells per well in 8-well IBIDI chamber slides and infected with 30,000 sporozoites per well of either *Py*WT or *Py*^*Pv*PUF1^. Cells were fixed at day 2 p.i. and processed for immunofluorescence.

### Live Imaging and Immunofluorescence assay.

Immunofluorescence. For *in vivo P. vivax*, livers were harvested from infected FRGN huHep mice on days 8 or 9 post-infection (p.i.), fixed in 4% (vol/vol) paraformaldehyde (PFA; Alfa Aesar) in 1× PBS, and sectioned into either 50-μm vibratome sections or 5-μm cryosections. Sections were blocked in normal goat serum (1:500) for 2 h, washed three times with PBS, and incubated overnight with anti-PfHSP70 (1:1,000; StressMarq, Cat# PC-186) together with either mouse or rabbit anti-PvUIS4 (1:1,000)^25^. After three PBS washes, sections were incubated with the appropriate fluorophore-conjugated secondary antibodies and DAPI for 2 h, washed three additional times with PBS, mounted in antifade mounting medium, and stored at 4 °C until imaging. Similarly, for *in vivo P. yoelii*, livers from mice co-infected with *Py*WT and *Py*^*Pv*PUF1^ were collected following liver perfusion on days 2, 3, 4, 5, and 6 p.i. and sectioned into 50-μm vibratome sections. Sections were processed as described above and stained with anti-PyUIS4 (1:1,000)^49^; and anti-mCherry (1:1,000; Invitrogen, Cat# MA5–15256), or anti-PvHSP60 (1:1,000)^16^; and anti-PyACP (1:500)^16^.

For *in vitro P. vivax*, primary human hepatocytes (Donor ESV) cultured in 384-well plates and infected with either PvSPZ (Chesson) or PvSPZ (Thai strain) were fixed on days 3, 5, 7, or 8 p.i. in 4% (vol/vol) PFA in 1× PBS. Cells were blocked in normal goat serum (1:500) for 1 h and incubated for 1 h with anti-PfHSP70 (1:1,000; StressMarq, Cat# PC-186), anti-PvUIS4 (1:500)^25^ and anti-PvLISP2 (1:500)^30^. Following three PBS washes, cells were incubated with the appropriate fluorophore-conjugated secondary antibodies and DAPI for 1 h, washed three times with PBS, and stored at 4 °C until imaging. Similarly, *in vitro P. yoelii*-infected HepG2-CD81 cells cultured in 8-well chamber slides (Ibidi Cat# 80809) were fixed on day 2 p.i. in 4% (vol/vol) PFA in 1× PBS and stained with anti-PyUIS4 (1:1,000)^49^; anti-PvHSP60 (1:1,000)^16^; PMID:), anti-PyACP (1:500)^16^, anti-PyMSP1 (1:250)^50^, or anti-mCherry (1:1,000; Invitrogen, Cat# MA5–15256) using the same protocol described above. All primary antibodies were diluted in 1× PBS containing 1% (w/v) BSA and 0.2% Triton X-100. Alexa Fluor 488-, 594-, and 647-conjugated donkey anti-mouse, anti-rabbit, anti-rat, and anti-goat secondary antibodies (Invitrogen) were used according to the manufacturer’s recommendations.

RNA fluorescence in situ hybridization (RNA-FISH) combined with immunofluorescence assay (IFA): The RNAscope Multiplex Fluorescent Assay v2 (Advanced Cell Diagnostics) was carried out according to the manufacturer’s instructions on *Pv* liver-stage schizonts and hypnozoites derived from infected primary human hepatocytes (BioIVT, batch #ESV) at day 8 p.i.. Cells were treated with protease Solution 3 (1:10 dilution) for 20 minutes at room temperature, followed by overnight incubation with mouse monoclonal anti-PvUIS4 antibody (1:1000 dilution)^25^ and fixed in neutral-buffered formalin for 10 minutes.

Probe hybridization was performed for 2 hours at 40 °C using probes targeting *Pv* transcripts PVP01_1015200 (PUF1) (20ZZ probe, regions 772–1858) and PVP01_0304700 (LISP2) (20ZZ probe, regions 2–1057). Signal amplification was achieved using tyramide signal amplification (TSA) with TSA Vivid fluorophores, following the manufacturer’s protocol. Cells were counterstained with Alexa Fluor 488-conjugated secondary antibody and DAPI.

Imaging was performed using the Stellaris 8 confocal microscope (Leica Microsystems), with image processing via Leica Lightning and analysis conducted in Fiji (ImageJ).

### RNA-seq library preparation

For *Pv* RNA-seq library preparation, total RNA was extracted from FRGN huHep mouse livers infected with 600,000 field-strain Pv SPZ and either left untreated or treated orally with PI4K inhibitor (30 mg/kg, days 4–7 post-infection). RNA was isolated using TRIzol (Thermo Fisher) and purified with the RNeasy Mini Kit (Qiagen) following the manufacturers’ instructions. A SureSelect XT custom oligo library was designed with Agilent (Design ID: S0782852) to enrich for Pv-specific complementary (c)DNA among the pool of human, mouse and parasite cDNA obtained from the RNA extraction from the humanized mouse liver. A total of 85,000 probes of 120-bp size were tiled every 100 bp across the entire Pv Sal I genome. Sequences >30% similar to human sequences were excluded. Sequencing libraries were prepared according to the SureSelect XT RNA Target Enrichment for Illumina Multiplexed Sequencing protocol from Agilent (Ref: 5190-4393). Libraries were analysed using a BioAnalyzer and quantified using qPCR. Illumina libraries were sequenced on Mi-seq as 75-bp single-end reads. PyGFP-infected primary hepatocytes and PyGFP-infected HepG2-CD81 cells were isolated at 36 h p.i. for fluorescence-activated cell sorting (FACS). For primary hepatocytes, BALB/cByJ mice were intravenously injected with 2 million PyGFP sporozoites. At 36 h p.i., hepatocytes were isolated by liver perfusion and digestion as previously described^29^. Briefly, mice were deeply anesthetized with ketamine (100 mg kg–1 body weight) and xylazine (10 mg kg–1 body weight). Livers were perfused with perfusion buffer I (0.5 mM EGTA in DPBS without Ca^2+^ and Mg^2+^) and subsequently digested with perfusion buffer II (50 μg ml–1 Liberase TL and 800 μM CaCl_2 in DPBS). Hepatocytes were dispersed in complete DMEM and centrifuged twice at low speed (50 × g, 2 min, 10 °C). For HepG2-CD81 infections, 1 million cells were seeded in collagen type I-coated 6-well plates and maintained in complete DMEM supplemented with 10% FBS, 1% penicillin-streptomycin, and 1% GlutaMAX. The following day, cells were infected with 800.000 PyGFP sporozoites per well. Cultures were washed at 3 h p.i. to remove extracellular and non-invading sporozoites and again at 24 h p.i. with complete medium. At 36 h p.i., cells were washed three times with PBS and detached using 500 μl of 0.25% trypsin-EDTA (Gibco) for 2 min. Trypsinization was stopped with 1.5 ml of complete medium, and cells were washed three times with PBS. The resulting cell pellets were resuspended in complete medium, and cell viability was assessed by trypan blue exclusion. Cell suspensions were adjusted to a final concentration of 2 × 106 cells ml–1 and processed for FACS. Approximately 10,000 GFP-positive cells were sorted directly into QIAzol lysis buffer for downstream RNA extraction. RNA-seq libraries preparation, total RNA was extracted from sorted infected primary human hepatocytes using miRNeasy micro kit (QIAGEN) according to manufacturer instructions, including on-column DNase digestion. Libraries were prepared using SMART-seq v.4 Ultra Low Input (Clontech) and were sequenced on the Illumina NextSeq 500 as 75-bp paired-end reads. The resulting data were demultiplexed using bcl2fastq2 (Illumina) to obtain fastq files for the downstream analysis. A minimum of 3 biological replicates were analysed.

### RNA-seq analysis

RNA-seq data were processed with the nf-core/rnaseq v3.22.2 pipeline^51^ using STAR 2.7.11b^51^ for genome alignment and Salmon 1.10.3^52^ for transcript quantification. Genes in the *P. vivax* RNA-seq experiment were filtered to those with over 20 counts-per-million (CPM) in at least three samples, leaving 3326 genes. The Bioconductor package edgeR^53^ was used to normalize library sizes using the TMM method, estimate dispersion, and test for differential gene expression between PI4K and controls using the exact test.

### Plasmid Construction

The parental plasmid used in this study was generated and described previously^22^. This vector contains the 5′ and 3′ homologous flanking regions of the *Py*P230P locus for targeted genomic integration, the uis4 promoter together with its corresponding 3′ regulatory/terminator sequence, and an mCherry fluorescent reporter cassette. A codon-optimized open reading frame (ORF) encoding PvPUF1 (GENEWIZ) was subsequently inserted into the vector via the PmeI and KpnI restriction sites. This cloning strategy generated the final construct, pUIS4-PvPUF1-mCherry (Extended data Figure 4a).

### Generation of *Plasmodium yoelii* parasites expressing *Pv*PUF1

Parasite transfection and clonal selection were performed as previously described^22^. Briefly, plasmid DNA was purified using a QIAGEN Plasmid Midi Kit (Cat. No. 12143) and quantified prior to transfection. For each transfection, 10 μg of plasmid DNA was resuspended in 100 μL of Human T Cell Nucleofector Solution (Lonza, Cat. No. VPA-1002). Highly synchronized blood-stage schizonts of the P. yoelii XNL strain were enriched by density-gradient centrifugation using 60% Accudenz. Purified schizonts were washed once with incomplete RPMI medium and resuspended in nucleofection buffer containing the plasmid DNA. Electroporation was carried out using an Amaxa Nucleofector II device (Lonza) with program U-033. Following nucleofection, transfected parasites were immediately recovered in 100 μL pre-warmed complete RPMI medium prior to inoculation into mice. For parasite recovery and propagation in vivo, the entire transfection mixture was administered to anesthetized Swiss Webster mice via retro-orbital injection. Drug selection was initiated 24 h post-transfection by providing pyrimethamine (7 mg/L) in the drinking water and was maintained until drug-resistant parasites became detectable, typically within 3–5 days. To establish clonal parasite populations, limiting-dilution cloning was performed. Infected blood was serially diluted with naïve erythrocytes to achieve an inoculum containing approximately three parasites per mouse and subsequently administered via retro-orbital injection into recipient mice. Clonality was confirmed by genotypic analysis. Using a CRISPR/Cas9-mediated genome-editing approach, we successfully generated the transgenic parasite line *Py*^*Pv*PUF1^. All oligonucleotide primers used for genomic screening of the *Py*^*Pv*PUF1^ parasite line are (put primers here). All primers were synthesized by Integrated DNA Technologies (IDT).

### Predicted PUF1 targets

All orthologs of *P. vivax* PUF1 (gene id PVP01_1015200) and Toxoplasma gondii PUF1 (TGME49_260600) were downloaded from PlasmoDB and ToxoDB, respectively^54^. Pumilio proteins from *Homo sapiens*, *Drosophila melanogaster*, and *Saccharomyces cerevisiae* were downloaded from UniProt, and were filtered to those most closely related to *P. vivax* PUF 1 after preliminary analysis (Q14671 and Q8TB72 in human, P25822 in *Drosophila*, P25339 in *Saccharomyces*). In total, 68 protein sequences representing PUF1 orthologs were aligned with Clustal Omega^55^. Within the alignment, positions of the nine alpha helices that are expected to face the RNA molecule were identified by visual inspection of a model of *P. vivax* PUF1 generated by AlphaFold 3^56^. Eight of these coils corresponded to canonical Pumilio repeats, within which we identified the 24 residues that form the “code” that controls the RNA sequence bound by the protein^57^. These 24 residues within all *Plasmodium* and *Toxoplasma* PUF1 orthologs were an exact match for *Saccharomyces* PUF4 (P25339), suggesting that the protein binds the RNA motif UGUANNNUA^33^. To predict transcripts containing the UGUANNNUA motif, the *P. vivax* P01 and *P. yoelii* 17X gene annotations from PlasmoDB 68 were modified to add 5’ and 3’ UTRS to any genes that didn’t have them, with a length of 808 and 688 respectively since these were the median annotated UTR widths in *P. vivax* P01. Motifs were only considered to be located within a gene if they were in the correct orientation, and were identified separately within 5’ UTR, spliced CDS, and 3’ UTR regions. The UGUANNNUA motif was found 7272 times within *P. vivax* transcripts and 13,304 times within *P. yoelii* transcripts, with lower GC content in *P. yoelii* as compared to *P. vivax* accounting for the greater frequency in *P. yoelii*. Each of the 64 possible UGUANNNUA sequences was tested for enrichment within transcript sequences. The 5’ UTR, CDS, and 3’ UTR regions were each tested separately for each species (*P. vivax* or *P. yoelii*). Nucleotide frequencies were calculated within each region type and species, and from these the expected rate of the motif under random chance was calculated. A one-sided Poisson test was then performed to see if the observed count of a given motif within a given species and region type was higher than expected. The UGUACAUUA and all UGUAWWWUA motifs were significant at a global FDR < 0.05 in both 5’ UTRs and 3’ UTRs of *P. vivax*, and were treated as putative PvPUF1 binding sites in all downstream analysis (Extended data Figure 5c). Using the log-fold-change values returned by the exact test for differential gene expression between *P. vivax* hypnozoites and schizonts, two-sided Student’s t-tests were performed to see if transcripts with a given putative PvPUF01 binding motif had a different distribution of log-fold-change values than those without it. To test for overlapping gene expression between *P. vivax* and *P. yoelii* schizonts, the three control *P. vivax* samples, and the three *P. yoelii* samples at the 36-hour timepoint were used. At 1 CPM, 4568 genes were expressed in all three samples of *P. vivax* and 4347 in all three samples of *P. yoelii*. Across these two sets of genes, there were 3511 orthologous pairs as listed in PlasmoDB^54^.

### Gene ontology overrepresentation

Across the 3511 pairs of orthologous genes considered to be expressed in *P. vivax* and *P. yoelii*, the UGUACAUUA or UGUAWWWUA motif was detected in 649 *P. vivax* genes and 2437 *P. yoelii* genes. Gene ontology (GO) terms, and their mappings to *P. vivax* and *P. yoelii* genes, were downloaded from PlasmoDB. Human-curated GO term mappings only were used, ignoring mappings from computational predictions. Overrepresentation of GO terms within genes with the UGUACAUUA or UGUAWWWUA motif was performed using the fgsea Bioconductor package^58^.

### GeoMx

Slides preparation: Cryosections of FRGN HUHEP mice infected with cryopreserved *Pv* Chesson SPZ. Sections were backed at 37°C for 1hour and then fixed overnight in 10%NBF at 4C. Then section was then treated with a diluted proteinase K solution (0.1 μg/mL). The tissue was then stained with anti-CK18/8 (1:500), anti-hFAH (1:500), anti-HSP70 (1:1000), sections were then hybridizated with the GeoMx WTA probes overnight following manufacturer instructions.

Data analysis: All 12 ROIs passed GeoMx DSP quality control after applying a limit of quantification (LOQ) threshold defined from negative-control probes. ROI segments were retained if they exceeded minimum sequencing depth (~1,000 raw reads), showed acceptable alignment, trimming, and stitching rates (~80%), and achieved sufficient sequencing saturation (>50%). Negative probe counts and NTC values were inspected to confirm low background and absence of contamination, and nuclei counts and ROI areas were reviewed to ensure consistent tissue sampling. After LOQ filtering, 3,086 human transcripts were carried forward for analysis. Data were normalized using the 75th-quantile (Q3) method, which correlated strongly with the geometric mean of negative-control probe counts. Gene-level variability was assessed by calculating the coefficient of variation (CV = SD/mean). Pathway analysis was performed using KEGG BRITE–defined gene sets, and Gene Set Variation Analysis (GSVA) was used to generate pathway enrichment scores for all ROIs containing at least five but fewer than 500 genes per gene set.

## Supplementary Material

This is a list of supplementary files associated with this preprint. Click to download.

- 05Table1PvivaxPyoeliiTranscriptome.xlsx
- 06Table2Motifanalysis.xlsx
- 07Table3GeoMx.xlsx
- 03ExtendedDataFigures.pdf
- 04ExtendedData1.pdf

EXTENDED DATA

**Extended_data_Figure1: Production of PvSPZ (Chesson) and in vivo infection workflow. (a),** Schematic overview of the PvSPZ (Chesson) production pipeline **(b),** Experimental workflow for FRGN huHep mouse infections. Created with BioRender.com.

**Extended_data_Figure2: *P. vivax* Chesson liver-stage development in primary human hepatocytes. (a),** Representative bright-field live images of the different PHH donors BGW, ESV, HTL, and ONR, 8 days post seeding. Scale bars, 50 μm **(b),** Quantification of total liver-stage parasites (schizonts, blue; hypnozoites, red) across donors at day 5 p.i. **(c),** Ratio of hypnozoites to schizonts per donor at day 5 p.i.

**Extended_data_Figure3: (a),** Heatmap showing expression of the top differentially expressed genes identified in kinase inhibitor-treated (PI4K) and control (Mix) *P. vivax* liver-stage samples. Expression values are displayed as row Z-scores of log2(CPM + 1)**. (b),** Gene Ontology enrichment analysis of genes upregulated following kinase inhibitor treatment. Bubble size indicates the number of genes assigned to each category and color represents enrichment significance (−log10 adjusted *P* value). **(c)**, Representative well from a 384-well plate of ESV primary human hepatocytes infected with the PvSPZ (Thai) field isolate VTTY320. *Pv*PUF1 and *Pv*LISP2 transcripts were detected by RNA-FISH (yellow and red, respectively), together with immunofluorescence staining for *Pv*UIS4 (green) and DAPI nuclear counterstaining (blue). Growth area per well 10 mm^2^. Inset shows a magnified view of hypnozoites. Scale bar, 100 μm. **(d),** Representative images of *Pv* Chesson and Thai field isolate VTTY297 schizonts and hypnozoites in ESV primary human hepatocytes stained with DAPI (blue), α-UIS4 (green), and α-LISP2 (red). Scale bars, 10 μm. **(e),** Quantification of PUF1 RNA-FISH, LISP2 RNA-FISH, and LISP2 protein expression in *Pv* Chesson and Thai field-isolate liver stages. Percentages indicate the proportion of parasites positive for each marker. Sample sizes are indicated for each parasite population.

**Exteded_data_Figure4**: **Generation and characterization of the *P. yoelii Pv*PUF1 overexpression parasite line. (a),** Schematic of the CRISPR/Cas9-mediated strategy used to generate the *Py*^*Pv*PUF1^ parasite line. Primer positions used for diagnostic PCR are indicated. **(b),** Quantification of blood-stage parasitemia, exflagellation center formation, oocyst numbers per mosquito midgut, and salivary gland sporozoite numbers in PyXNL and *Py*^*Pv*PUF1^ parasites. Each symbol represents an independent biological replicate. Statistical significance was assessed using Welch’s t-tests and two-way ANOVA, as indicated. ns, not significant. **(c),** Immunofluorescence analysis of *Py*WT and *Py*^*Pv*PUF1^ liver-stage parasites developing in BALB/cByJ mice at 44 h p.i.. Parasites were stained with antibodies against mCherry (red), the apicoplast marker ACP or the mitochondrial marker HSP60 (green), and UIS4 (yellow), as indicated. Nuclei were counterstained with DAPI. Scale bars, 10 μm. **(d),** Immunofluorescence analysis of *Py*WT and *Py*^*Pv*PUF1^ liverstage parasites in HepG2-CD81 cells at 44h p.i.. Parasites were stained with antibodies against mCherry (red), the maturation marker MSP1 and the endoplasmic reticulum marker BiP (green), and HSP70 (yellow), as indicated. Nuclei were counterstained with DAPI. Scale bars, 10 μm.

**Extended_data_Figure5: Prediction and comparative analysis of *Pv*PUF1-associated RNA motifs. (a),** AlphaFold structural predictions of *Pv*PUF1 RNA-binding domain **(b),** AlphaFold structural predictions of *Py*PUF1 RNA-binding domain. **(c)** Enrichment analysis of predicted PUF1-associated motifs across 5′UTRs, coding sequences (CDS), and 3′UTRs of *Pv* and *Py* liver-stage transcripts. The y-axis indicates the rate ratio derived from the Poisson enrichment analysis. Significantly enriched motifs following multiple-testing correction (FDR < 0.05) are highlighted in red. Labels in red indicate the three nucleotides within the variable region of the UGUANNNUA motif. **(d)** Ranked distribution of differential expression values for *P. vivax* liver-stage transcripts. Transcripts containing the UGUACAUUA motif within the 3′UTR are highlighted in red and labeled. **(e)** Venn diagram showing the overlap of orthologous genes expressed in the liver-stage transcriptomes of *Pv* and *Py* used for comparative motif analyses.

**Extended_data_Figure6: (a),** Sequencing metrics across all Areas of Interest (AOIs): raw, trimmed, stitched, aligned, and deduplicated reads. Consistent read depth across ROIs indicates high-quality spatial profiling. (**b),** Heatmap of GSVA z-scores across spatial transcriptomic ROIs annotated by parasite status (PVHYP, PVSCH, Uninfected). **(c)** Volcano plots summarizing significantly enriched pathways across spatial transcriptomic ROIs. From left to right: Schizont vs Uninfected, Hypnozoite vs Uninfected, and Hypnozoite vs Schizont. Labeled genes represent top significant hits (FDR < 0.001 and |log_2_FC| > 0.5). Grey points denote non-significant pathways; orange-highlighted points indicate significant pathways (FDR < 0.05)

Extended_data_1

Table_1_*Pvivax_P.yoelii_*RNAseq

Table_2_Motif_Analysis

Table_3_GeoMx

## Figures and Tables

**Figure 1:**
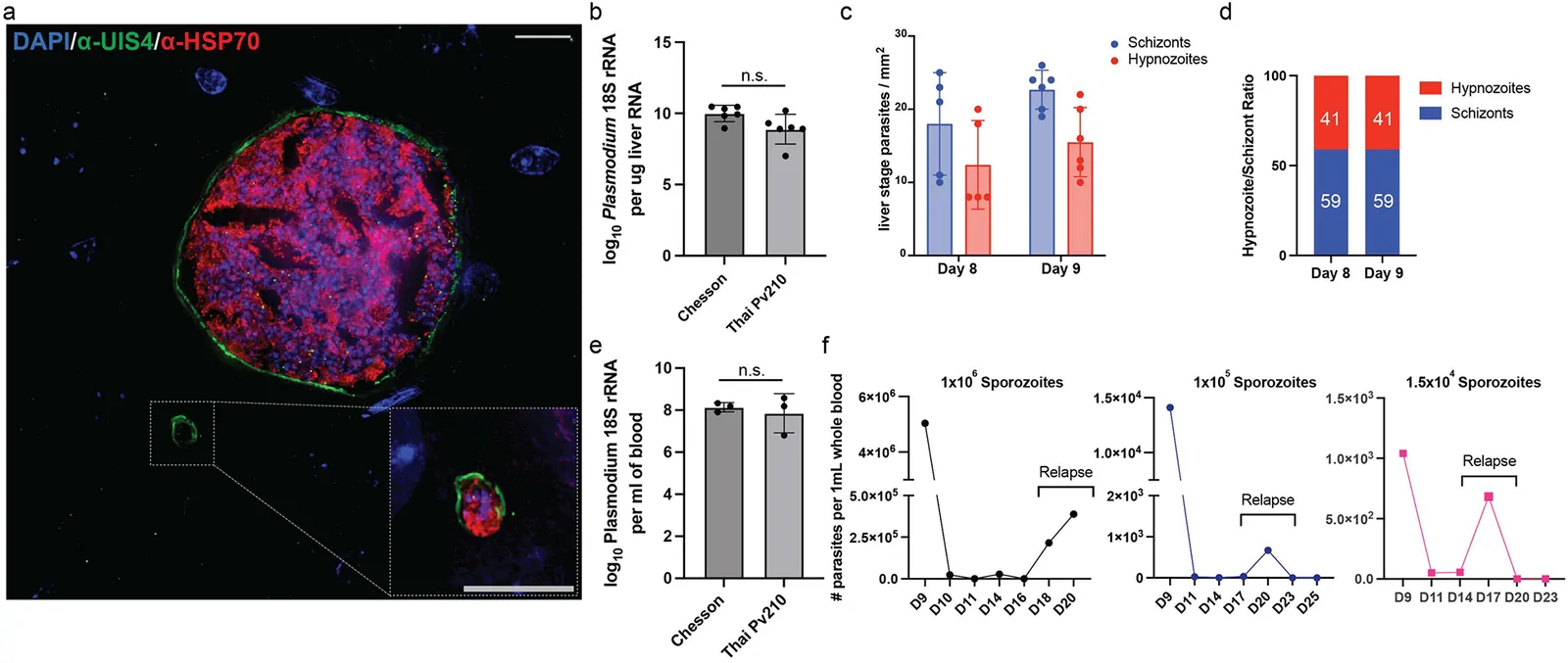
Cryopreserved PvSPZ (Chesson) establish liver infection and relapse in the FRGN huHep mouse model: **(a)**, High-resolution image illustrating hypnozoite and schizont morphology at day 9 pi. Parasites are delineated by UIS4 (green) and HSP70 (red) with host and parasite nuclei counterstained with DAPI (blue) Scale bars: 10 μm (main), 5 μm (insert). **(b)**, Liver parasite quantification by qRT-PCR of *P. vivax* 18S rRNA at day 8 pi following infection with cryopreserved PvSPZ (Chesson) versus freshly isolated Thai/VK210 PvSPZ. **(c)**, Quantification of hypnozoites and schizonts in liver lobes at days 8 and 9 pi. Bars represent the hypnozoite and schizont counts (**d**), Proportion of hypnozoites and schizonts at days 8 and 9 pi. (**e**), Detection of *P. vivax* 18S rRNA in the mouse blood at day 9 pi, marking liver egress of exo-erythrocytic merozoites. (**f**), Relapse kinetics following infection with 1,000,000 (left), 100,000 (middle), or 15,000 (right) PvSPZ (Chesson). Relapses were measured by qRT-PCR.

**Figure 2:**
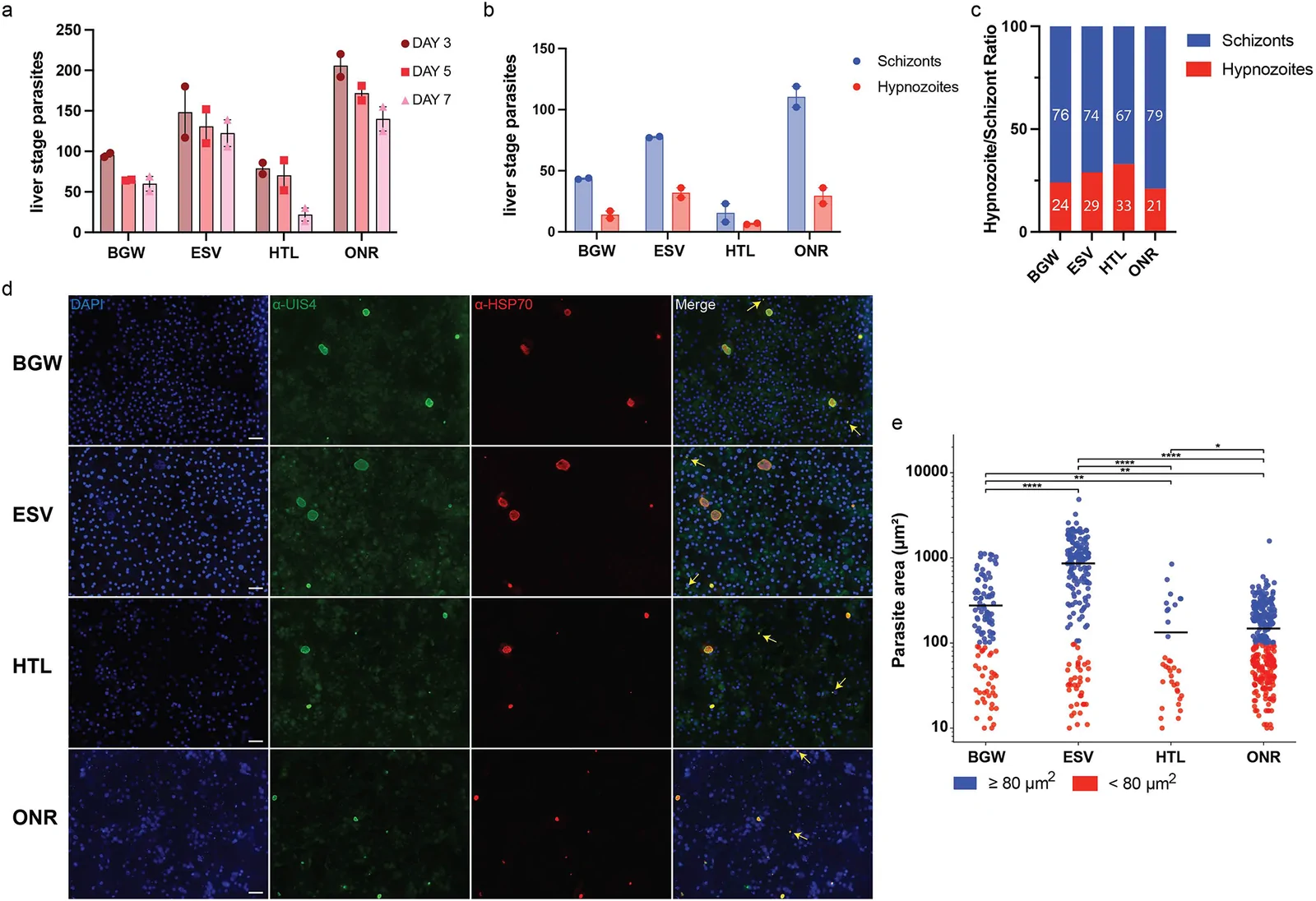
Cryopreserved PvSPZ (Chesson) infect primary human hepatocytes and generate both schizonts and hypnozoites. **(a),** Total number of *Pv* liver-stage parasites quantified at days 3, 5, and 7 p.i. across four primary human hepatocyte donors (BGW, ESV, HTL, ONR). (**b**), Day-7 quantification of schizonts and hypnozoites in each donor. (**c**), Hypnozoite-to-schizont ratios at day 7 p.i. (**d**), Representative immunofluorescence images across donors of liver-stage parasites stained with DAPI (nuclei), α-UIS4 (green), and α-HSP70 (red). Scale bar 50 μm. (**e**), Distribution of liver stage areas, in each donor at day 7 p.i.. Horizontal bars indicate group means. Statistical comparisons were performed using two-sided Mann–Whitney U tests with Benjamini–Hochberg FDR correction for multiple pairwise comparisons. P < 0.05, *P < 0.01, **P < 0.001, ***P < 0.0001. Red points indicate parasites <80 μm^2^; blue points indicate parasites ≥80 μm^2^.

**Figure 3:**
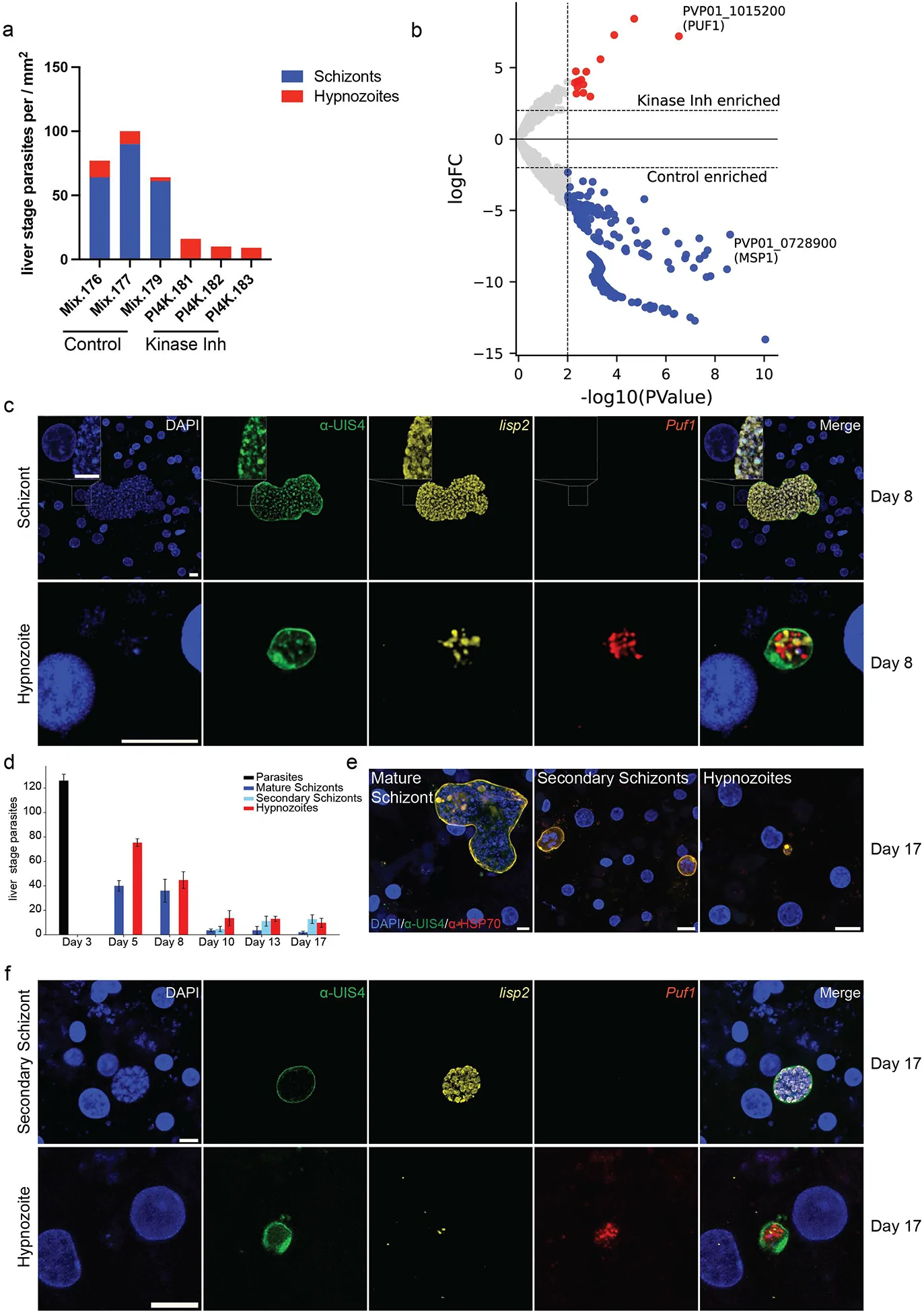
PUF1 distinguishes dormant hypnozoites from replicating schizonts. **(a),** Quantification of liver-stage parasites per mm^2^ in FRGN huHep mice infected with *Pv* field isolates SPZ, untreated (Mix-176, Mix-177, Mix-179) or PI4K-treated animals (PI4K-181, PI4K-182, PI4K-183). Schizonts (blue) and hypnozoites (red). (**b**) Volcano plots show transcripts differentially expressed between mix population (untreated) and hypnozoite-enriched (PI(4)K-treated) samples. Labeled genes represent top significant hits (FDR < 0.001). Grey points denote non-significant genes; **(c),** RNA-FISH validation of PUF1 expression *in vitro* in primary human hepatocyte (ESV) infected with PvSPZ (Chesson). Representative images show RNA-FISH probes *PvLISP2* (yellow), *PvPUF1* (red), IFA *Pv*UIS4 (green), and DAPI (blue). Scale bars: 10 μm **(d),** Temporal dynamics of liver-stage development across days 3 to 17 p.i., quantifying total parasites, mature schizonts, secondary schizonts, and hypnozoites. **(e)**, Representative day-17 p.i. images show morphology of mature schizonts, secondary schizonts, and hypnozoites stained with DAPI, anti-UIS4, and anti-HSP70. Scale bars, 20 μm. **(f**) RNA-FISH analysis at day 17 p.i.. RNA-FISH probes *PvPUF1* (yellow), *PvLISP2* (red), IFA UIS4 (green), and DAPI (blue). Scale bars: 10 μm

**Figure 4:**
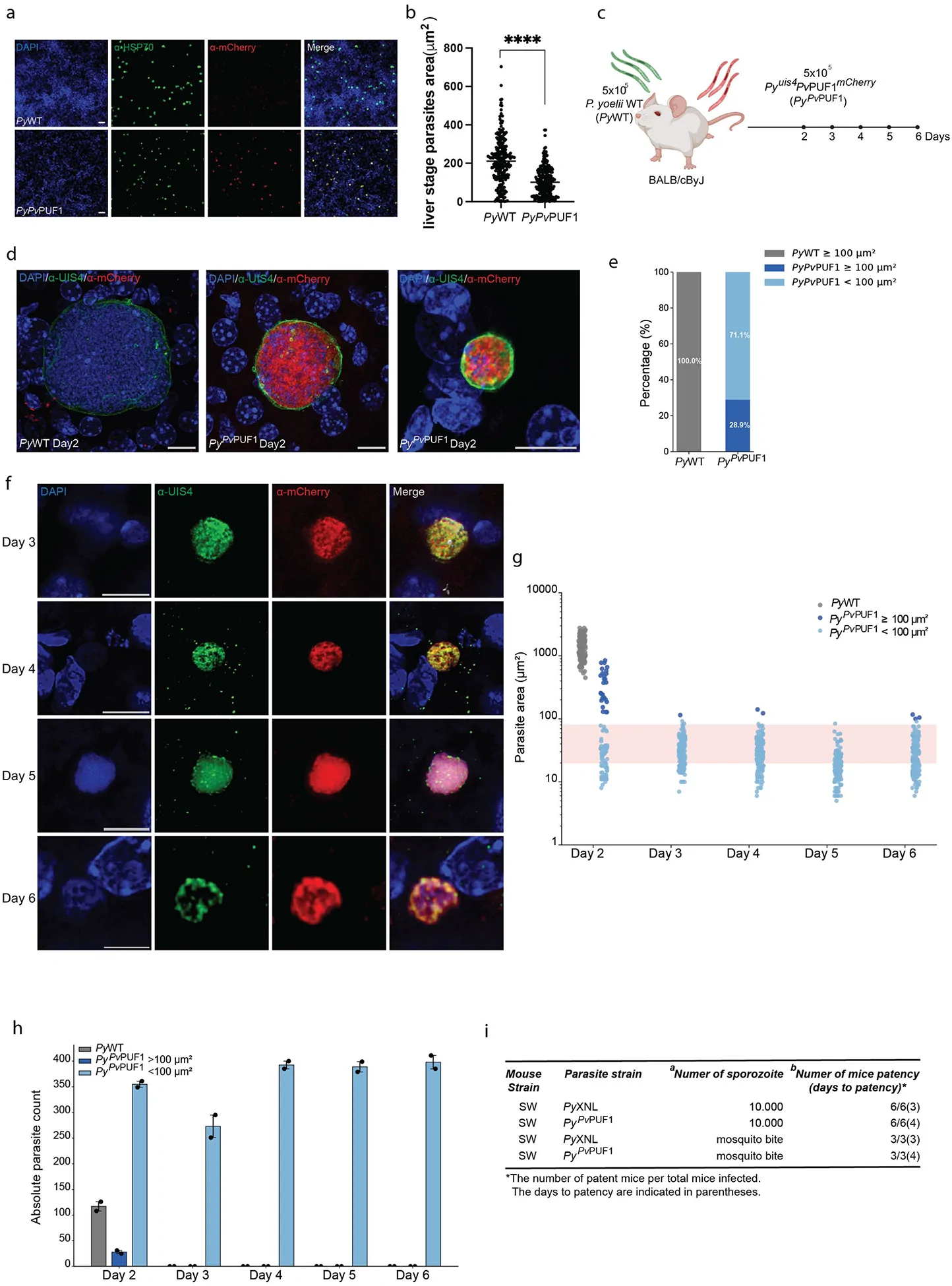
Heterologous expression of *Pv*PUF1 in *P. yoelii* induces persistent growth-restricted liver stages. **(a),** Representative immunofluorescence images of *P. yoelii* wild-type (PyWT) and transgenic *Py*^*PvPUF1*^ liver-stage parasites in infected HepG2-CD81 stained with DAPI (blue), α-HSP70 (green), α-mCherry (red), and α-ACP (yellow) at 44h p.i.. Scale bars, 50 μm. **(b),** Quantification of liver-stage parasite area in *Py*WT and *Py*^*Pv*PUF1^ parasites at day 2 post-infection. Each dot represents an individual parasite. Statistical analysis performed using Mann–Whitney test; ****P < 0.0001. **(c),** Experimental schematic of BALB/cByJ mouse infections with *Py*WT or transgenic *Py*^*PvPUF1*^ sporozoites expressing *Pv*PUF1 under the control of the UIS4 promoter. Liver-stage infections were analyzed from days 2–6 p.i.. Created with BioRender.com. **(d),** Representative immunofluorescence images showing the *Py*WT schizont and the two types of *Py*^*Pv*PUF1^ parasites at day 2 p.i., stained with DAPI (blue), α-UIS4 (green), α-mCherry (red), Scale bar, 10 μm. **(e)** Stacked bar plot showing the relative proportion of parasite populations based on size distribution at day 2 p.i.. **(f),** Representative immunofluorescence images of persistent *Py*^*Pv*PUF1^ liver stages from days 3–6 p.i. stained with DAPI (blue), α-UIS4 (green), and α-mCherry (red). Scale bar, 5 μm. **(g),** Quantification of parasite area during liver-stage development from days 2–6 p.i.. Different colors represent different parasite size. Red ribbon indicates *Pv* hypnozoite size range. **(h),** Quantification of liver-stage parasite populations during infection. **(i),** Blood-stage patency following intravenous sporozoite injection or mosquito bite infection with *Py*WT or *Py*^*Pv*PUF1^ parasites. Numbers indicate patent mice over total infected mice, with days to patency shown in parentheses.

**Figure 5:**
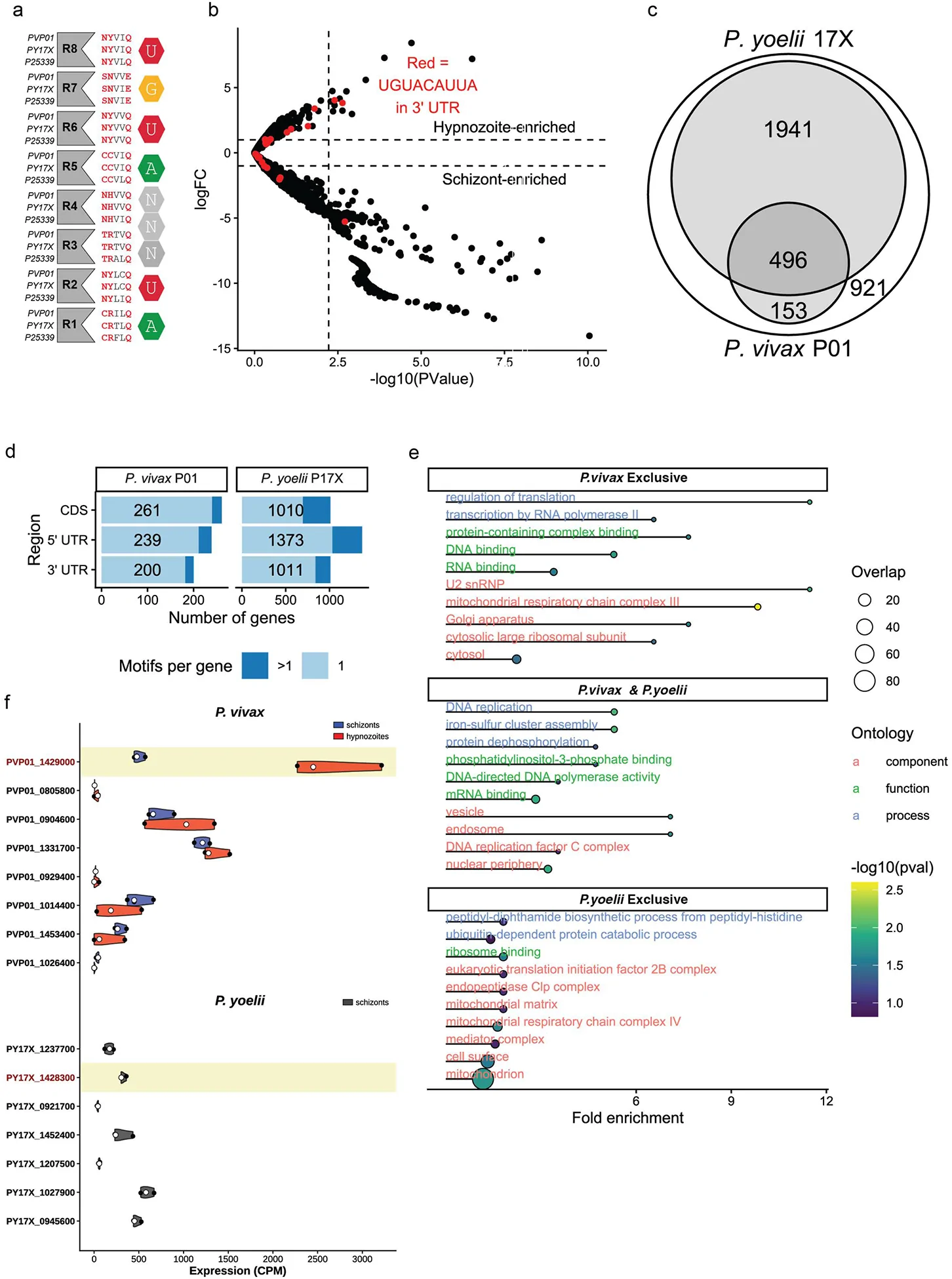
Comparative motif analysis reveals conserved PUF1-associated transcript networks in *P. vivax* and *P. yoelii* liver stages. **(a),** Conservation of amino acid residues predicted to mediate RNA recognition across *Pv*PUF1 (PvP01), PyPUF1 (Py17X), and *Saccharomyces cerevisiae* PUF4 (P25339). **(b),** Volcano plot showing differential expression of *Pv* liver-stage transcripts between hypnozoites and schizonts. The x-axis indicates −log10(P value) and the y-axis indicates log2 fold change. Transcripts containing the UGUACAUUA motif within the 3′UTR are highlighted in red. **(c),** Venn diagram showing the distribution of orthologous genes containing UGUAWWWUA-family motifs and/or UGUACAUUA among 3,511 genes expressed in the liver-stage transcriptomes of both *Pv* and *Py*. Numbers indicate how many transcripts contain a motif in one, both, or neither species **(d),** Distribution of motif-containing orthologous transcripts across 5′UTRs, coding sequences (CDS) and 3′UTRs in *Pv* and *Py*. **(e),** Gene Ontology overrepresentation analysis of motif-containing genes unique to *Pv*, unique to *Py*, or shared between both species. Bubble size represents the number of genes assigned to each category and color indicates enrichment significance (−log10 adjusted P value). **(f),** Expression of the CCR4–NOT complex components in *Pv* hypnozoites (red) and schizonts (blue) and *Py* liver stages (gray). Transcript abundance is shown as counts per million (CPM). CAF1 orthologues are highlighted.

**Figure 6.**
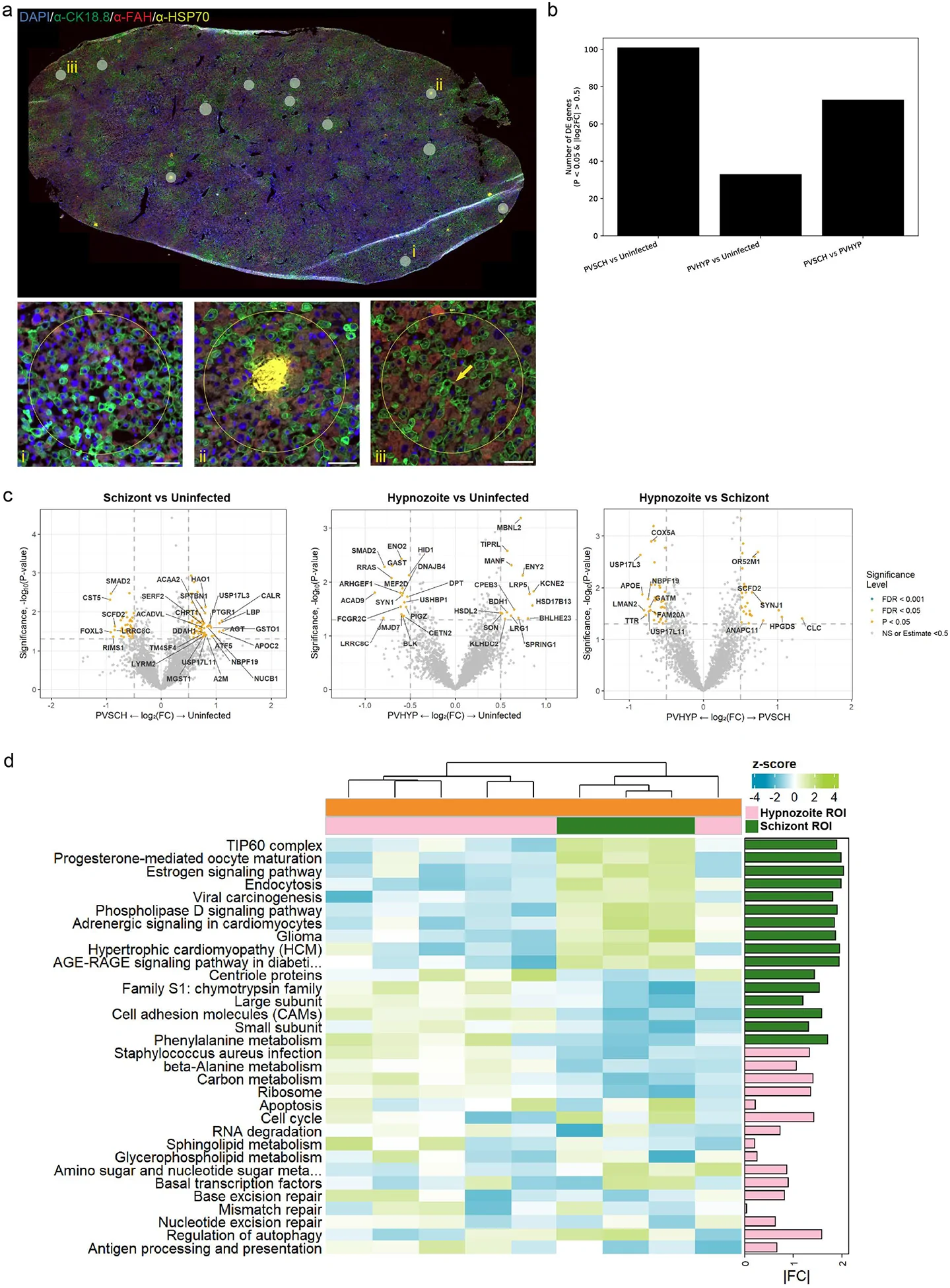
Spatial transcriptomic profiling of schizont- and hypnozoite-infected hepatocytes in the FRGN huHep mouse model. **(a)**, GeoMx DSP whole-slide image of PvSPZ (Chesson) infected FRGN huHep liver tissue at day 8 p.i. Sections were stained with DAPI (blue), anti-CK18/8 (green), anti-hFAH (red), and anti-HSP70 (yellow). The selected ROIs for spatial transcriptomic profiling are highlighted by circles. Representative high-magnification images of the selected ROIs containing uninfected cells (i) mature schizonts ROI (ii), and hypnozoites (iii) within their hepatic microenvironment. **(b),** Proportion of differentially expressed genes (DEGs) across comparisons: PVSCH vs Uninfected, PVHYP vs Uninfected, and PVHYP vs PVSCH. Bars indicate the percentage of genes meeting dual-significance thresholds (FDR < 0.05 and |log_2_FC| > 0.5). **(c),** Volcano plots summarizing differential expression across spatial transcriptomic ROIs. Labeled genes represent top significant hits (FDR < 0.001 and |log_2_FC| > 0.5). Grey points denote non-significant genes; orange-highlighted points indicate significant DEGs. **(d),** Pathway analysis Heatmap shows GSVA z-scores across significantly enriched KEGG pathways, clustered by parasite location. Bar plots (right) display log_2_ fold-change in pathway activity between Hypnozoite and Schizont segments.

## Data Availability

The GEO accession numbers for the *Pv* RNA-seq FASTQ files, *Py* RNA-seq FASTQ files and GeoMX data generated in this study are GSE220039, GSE337317, and GSE337318 respectively. Please refer to Supplementary Table 1 for associated sample identifiers; biological and technical replicate samples are identified by matching ‘RNA_label’.

## CODE AVAILABILITY

Computer code for PUF1 motif analysis is available on GitHub at https://github.com/RSC-RP/kappe_puf1_motifs_2026, archived on Zenodo at https://doi.org/10.5281/zenodo.21825603.
